# Putaminal Mosaic Visualized by Tyrosine Hydroxylase Immunohistochemistry in the Human Neostriatum

**DOI:** 10.3389/fnana.2016.00034

**Published:** 2016-04-05

**Authors:** Ryoma Morigaki, Satoshi Goto

**Affiliations:** ^1^Department of Neurodegenerative Disorders Research, Institute of Biomedical Sciences, Graduate School of Medical Sciences, Tokushima UniversityTokushima, Japan; ^2^Parkinson’s Disease and Dystonia Research Center, Tokushima University Hospital, Tokushima UniversityTokushima, Japan; ^3^Department of Neurosurgery, Institute of Biomedical Sciences, Graduate School of Medical Sciences, Tokushima UniversityTokushima, Japan

**Keywords:** putamen, matrix compartment, striosomes, tyrosine hydroxylase, human brain, immunohistochemistry

## Abstract

Among the basal ganglia-thalamocortical circuits, the putamen plays a critical role in the “motor” circuits that control voluntary movements and motor learning. The human neostriatum comprises two functional subdivisions known as the striosome (patch) and matrix compartments. Accumulating evidence suggests that compartment-specific dysregulations of dopamine activity might be involved in the disease-specific pathology and symptoms of human striatal diseases including movement disorders. This study was undertaken to examine whether or how striatal dopaminergic innervations are organized into the compartmentalized architecture found in the putamen of adult human brains. For this purpose, we used a highly sensitive immunohistochemistry (IHC) technique to identify tyrosine hydroxylase (TH; EC 1.14.16.2), a marker for striatal dopaminergic axons and terminals, in formalin-fixed paraffin-embedded (FFPE) tissues obtained from autopsied human brains. Herein, we report that discrete compartmentalization of TH-labeled innervations occurs in the putamen, as in the caudate nucleus (CN), with a higher density of TH labeling in the matrix compared to the striosomes. Our results provide anatomical evidence to support the hypothesis that compartment-specific dysfunction of the striosome-matrix dopaminergic systems might contribute to the genesis of movement disorders.

## Introduction

The human neostriatum comprising the caudate nucleus (CN) and putamen is the primary input region of the basal ganglia, a group of subcortical nuclei that control a wide range of sensorimotor, cognitive and emotional functions (for review see, Graybiel, 2008; Amemori et al., 2011). It has so far been known that the basal ganglia are organized into several structurally and functionally distinct neural circuits that link the cortex, basal ganglia, and thalamus, with each circuit focused on a different frontal cortex territory (Alexander et al., 1986, 1990). Among these basal ganglia-thalamocortical circuits, the putamen participates in the “motor” circuits and is thereby important to basal ganglia motor control and movement disorders (Albin et al., 1989; DeLong, 1990). Parkinson’s disease (PD) results from a striatal dopamine deficiency due to the neurodegenerative loss of dopamine-producing cells in the substantia nigra pars compacta (SNc; Hirsch et al., 1988). Kish et al. (1988) showed that the depletion of dopamine in patients with PD was most remarkable in the putamen, particularly in its caudal portions. Goto et al. (1989a, 1996) also documented that in parkinsonian patients, a pathological lesion was found specifically in the nigrostriatal loop that forms a link with the putamen.

The mammalian neostriatum is composed of two functional subdivisions known as the striosome (patch) and matrix compartments, which are developmentally, anatomically, and biochemically distinct (Graybiel and Ragsdale, 1978; Bolam et al., 1988; Graybiel, 1990; Gerfen, 1992). The striosome and matrix compartments make up about 15% and 85% of the volume of the striatum, respectively, in mammalian brains (Johnston et al., 1990). Although single dopaminergic afferent axons can terminate in both the compartments (Matsuda et al., 2009), the striosomes and matrix tend to receive dopaminergic afferents from distinct groups of SNc neurons. The striosomal cells are preferentially innervated by a group of dopamine-producing cells in the ventral tier of the SNc (Gerfen et al., 1987; Jimenez-Castellanos and Graybiel, 1987; Langer and Graybiel, 1989; Prensa and Parent, 2001), while the matrix cells preferentially receive the afferents arising from dopaminergic cells in the dorsal tier of the SNc, the ventral tegmental area and the retrorubral area (Gerfen et al., 1987; Jimenez-Castellanos and Graybiel, 1987; Prensa and Parent, 2001).

Accumulating evidence suggests that striosome-matrix dopaminergic systems exert a critical role in basal ganglia-thalamocortical circuits (Graybiel, 2008; Amemori et al., 2011). Interestingly, imbalanced dopamine activity between the striosome and matrix compartments has been suggested to underlie the genesis of pathological lesions and clinical symptoms that occur in multiple movement disorders (Graybiel, 2008; Goto et al., 2010; Crittenden and Graybiel, 2011), such as the L-DOPA-induced dyskinesia associated with PD (Graybiel et al., 2000). However, despite previous immunohistochemical studies using frozen sections (Ferrante and Kowall, 1987; Graybiel et al., 1987; Holt et al., 1997; Ciliax et al., 1999; Prensa et al., 1999, 2000), it remains undetermined whether or how striatal dopaminergic innervations might be organized into a discrete striosome-matrix axis in the human putamen.

To address this issue, we employed a highly-sensitive immunohistochemistry (IHC) method (Goto et al., 2015) to identify tyrosine hydroxylase (TH; EC 1.14.16.2) in formalin-fixed paraffin-embedded (FFPE) striatal tissues from autopsied human brains. TH serves as a reliable marker for visualizing dopaminergic axons and terminals in the striatum (Moss and Bolam, 2008). Here, we show that as in the CN, discrete compartmentalization of TH immunoreactivity occurs in the putamen, with a higher density of TH labeling in the matrix compartment relative to the striosomes. Our results provide anatomical evidence to support the hypothesis that compartment-specific dysfunction of the striosome-matrix dopaminergic systems might contribute to the genesis of human movement disorders.

## Materials and Methods

### Western Blot Analysis on Mouse Brains

All procedural protocols that involve mouse experiments were approved by the Ethical Review Committee of the Tokushima University. We used male C57BL/6 mice (Nihon SLC Co., Shizuoka, Japan; *n* = 3) aged 8–10 weeks. Western blot analysis was performed with a rabbit polyclonal antibody against TH (Sato et al., 2008), according to the method that we previously ported (Morigaki and Goto, 2015). Blots were developed by chemiluminescent autoradiography (ECL plus kit; GE Healthcare, Buckingham, UK).

### IHC for TH in Mouse Brains

After receiving an intraperitoneal injection of a lethal dose of pentobarbital (Sigma-Aldrich, St. Louis, MO, USA), mice (*n* = 5) were transcardially perfused with cold PBS, and followed by cold 4% paraformaldehyde in 0.1 M phosphate buffer (pH 7.2). Frozen sections with 16-μm thickness were processed for the IHC with the tyramide signal amplification (TSA) technique in a free-floating manner, as in our previous reports (Okita et al., 2012; Morigaki and Goto, 2015). Briefly, rabbit polyclonal antibody against TH (1:100,000) was used as a primary antibody. To detect the bound antibody, we used the Histofine Simple Stain Kit (Nichirei, Tokyo, Japan) and the TSA Plus Cyanine 3 System (Perkin Elmer, Shelton, CT, USA).

### Human Brain Tissue Preparation for IHC

All procedural protocols that involve autopsied human brains were approved by the Ethical Review Committee of the Tokushima University. FFPE striatal sections with 4-μm-thickness were prepared from the autopsied brain tissues of neurologically-normal patients (*n* = 5; mean age ± SEM, 59 ± 8 years), as we previously described (Morigaki and Goto, 2015). After deparaffinization and rehydration, all sections were treated with 1% H_2_O_2_ in water for 5 min. For the antigen retrieval, they were then immersed in 0.01 M sodium citrate buffer (pH 6.0) and placed in a 700-W microwave for 15 min. Endogenous binding sites for avidin and biotin were blocked by using the Avidin/Biotin Blocking Kit (Vector, Burlingame, CA, USA). The sections were then immersed in 3% BSA-PBS for 60 min and they were processed for the IHC protocols described below.

### IHC with 3,3′-Diaminobenzidine (DAB) in Human Brain Tissue

The sections were incubated for 18 h in 3% BSA-PBS containing a rabbit polyclonal antibody against TH (1:200,000; Sato et al., 2008) or [Met]-enkephalin (MEnk; 1:500,000; Millipore, St. Louis, MO, USA; Goto et al., 2015). They were then processed for a hybrid IHC protocol that implements aspects of both the polymer-staining and avidin-biotin-complex (ABC) methods combining with the biotin-TSA system, as we previously reported (Goto et al., 2015). Briefly, the sections were incubated for 30 min in the polymer-staining solution (Histofine Simple Stain Kit; Nichirei). After several rinses in PBS, they were incubated for 30 min with biotinyl TSA solution prepared from a TSA Biotin System kit (Perkin Elmer) with 1× Plus Amplification Diluent (Perkin Elmer). After several rinses in PBS, they were incubated for 30 min with the ABC reagent prepared from a Vectastain Elite ABC kit (Vector). After several rinses in PBS, the sections were immersed for 10 min in 0.05 M Tris-HCl (pH 7.4) containing 0.05% diaminobenzidine (DAB; Merck, Darmstadt, Germany) and 0.01% H_2_O_2_. After dehydration, the sections stained with DAB were cover-slipped with Malinol (Muto Pure Chemicals, Tokyo, Japan).

### IHC with Fluorescents in Human Brain Tissue

The sections were first incubated for 18 h in 3% BSA-PBS containing rabbit polyclonal antibody against TH (1:100,000; Sato et al., 2008). After several rinses in PBS, the bound antibodies were visualized by using the Histofine Simple Stain Kit (Nichirei) and the TSA Plus Cyanine3 System (Perkin Elmer). The sections were then treated for 30 min in 0.1 M glycine-HCl (pH 2.2) to remove the first primary antibodies. After several rinses in PBS, they were incubated for 18 h in 3% BSA-PBS containing rabbit polyclonal antibody against MEnk (1:200,000; Millipore) or dopamine and cAMP-regulated phosphoprotein of 32 kDa (DARPP-32; 1:20,000; Cell Signaling, Denver, MA, USA). The bound antibodies were visualized by using the Histofine Simple Stain Kit (Nichirei) and the TSA Plus Fluorescein System (Perkin Elmer; Morigaki and Goto, 2015).

### Digital Imaging and Morphometric Analysis

To capture macroscopic images, we used an Epson ES-2200 color image scanner (SEIKO EPSON Co., Nagano, Japan). Microscopic images were captured using an Olympus BX51 microscope (Olympus, Tokyo, Japan) equipped with a digital camera DP40 (Olympus). By means of Adobe Photoshop CS4, all captured images were digitally processed with minimal adjustments to contrast, brightness, and color balance.

Morphometric analysis was performed using an image-analyzer (MetaMorph, Molecular Device, Tokyo, Japan). We measured the optical density of TH-immunoreactive products in the striosome and matrix subfields represented by gray levels on non-colored digital images at low magnification, as previously reported (Sato et al., 2008; Morigaki and Goto, 2015). For each human striatum (*n* = 5), we measured five striatal subfields of the CN, rostral putamen, and caudal putamen.

We also measured the striosomal areas delineated by faint TH staining or dense MEnk staining in the CN (*n* = 5), rostral putamen (*n* = 5), and caudal putamen (*n* = 5) from each autopsied brain (*n* = 5). The mean values were used to quantify the percentage of the striatal area occupied by the striosome compartment.

To determine the cell density, we counted the numbers of neurons positive for TH or DARPP-32 in a 1 mm × 1 mm field in the human neostriatum, as described in our previous report (Goto et al., 2013). We examined five fields selected randomly in each of the CN and putamen from each patient (*n* = 5).

### Statistical Analysis

All quantitative data are presented as the mean ± SEM. Two-group comparisons were made using the Student’s *t*-test (two-tailed, paired). Significant differences were considered as *P* < 0.05.

## Results

### Immunochemical Detection of TH in Mouse Brains

**Figure 1:**
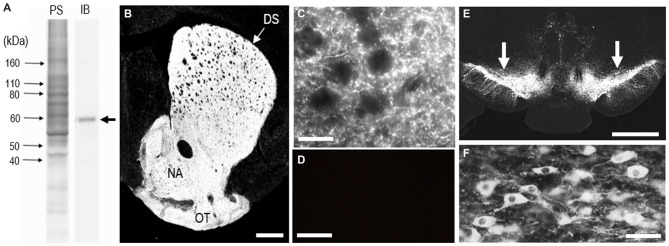
**Identification of tyrosine hydroxylase (TH) in the mouse striatum. (A)** Western blot assay. Crude homogenates of protein (10 μg) from the mouse striatum were separated on a 10% gel by SDS-PAGE and then immunoblotted using anti-TH antibody. An immunostained protein band (arrow) was selectively detected, with an approximate molecular mass corresponding to the predicted size of native TH protein. PS, protein staining; IB, immunoblot. **(B)** Photomicrograph of a striatal section stained for TH. TH immunoreactivity appears to be homogeneously distributed throughout the dorsal striatum (DS). **(C)** Photomicrograph of the striatal area stained for TH. Numerous tiny TH-positive dots were found in the striatum. **(D)** Photomicrograph of the DS processed using the immunostaining protocol without anti-TH antibody. **(E)** Photomicrograph of the midbrain stained for TH. Arrows indicate the substantia nigra pars compacta (SNc). **(F)** Photomicrograph of nigral dopaminergic cells positive for TH. Abbreviations: DS, dorsal striatum; NA, nucleus accumbens; OT, olfactory tubercle. Scale bars: **(B)** 500 μm, **(C,D)** 20 μm, **(E)** 1 mm, **(F)** 50 μm.

To determine the monospecificity of the anti-TH antibody used here, we first performed a western-blot analysis of the mouse brains. Immunoblots (IBs) of mouse striatal extracts showed a single protein band with an approximate molecular mass that corresponds to the predicted size of native TH protein (Figure 1A). The staining specificity was also assessed on frozen striatal sections from adult mouse brains with or without anti-TH antibody (Figures 1B–D). In the presence of the antibody, specific TH staining was detected in the striatum (Figure 1B), where numerous TH-positive puncta were densely found (Figure 1C). In agreement with our previous report (Sato et al., 2008), the distribution of TH labeling was diffuse and homogeneous throughout the striatum in adult mice. By contrast, TH immunoreactivity was not seen in striatal sections processed for the IHC protocol in the absence of the antibody (Figure 1D). In addition, strong TH labeling was also seen in the midbrain (Figure 1E), where SNc neurons exhibited intense TH immunoreactivity in their cell bodies and neurites (Figure 1F).

### Putaminal Mosaic Pattern Visualized by TH-Immunostaining with DAB in Human Brains

**Figure 2:**
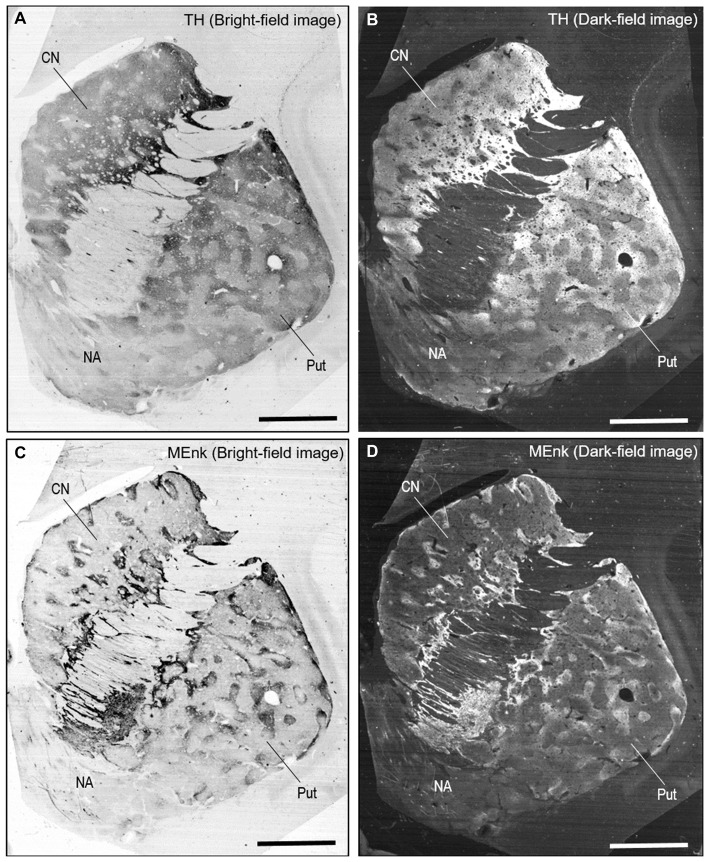
**Non-homogeneous distribution of TH in the human neostriatum. (A,B)** Bright-field **(A)** and dark-field **(B)** images taken from a frontal section of the striatum stained with diaminobenzidine (DAB) for TH. **(C,D)** Bright-field **(C)** and dark-field **(D)** images taken from a frontal section of the striatum stained with DAB for [Met]-enkephalin (MEnk), a marker for striosomes (patches). Note that they are contiguous to those shown in **(A,B)**. Abbreviations: CN, caudate nucleus; Put, putamen; NA, nucleus accumbens. Scale bars: 5 mm.

A highly-sensitive IHC technique (Goto et al., 2015) allowed us to detect TH immunoreactivity in FFPE human autopsy tissue. Specific TH labeling was identified in the neostriatum composed of the CN and putamen. No TH labeling was detected in striatal sections processed for the IHC protocol without the anti-TH antibody. Bright-field (Figure 2A) and dark-field (Figure 2B) images are shown of a striatal section stained with DAB for TH. These macroscopic images reveal that in both the CN and putamen, TH labeling is distributed in a non-homogeneous pattern of low- and high-imunoreactive zones. Notably, patches of low TH immunoreactivity were evident in the putamen, as in the CN.

On FFPE striatal tissue obtained from autopsied human brains, MEnk is one of the most reliable IHC markers for visualizing striatal compartments with higher density of MEnk labeling in the striosomes than in the matrix (Goto et al., 2013, 2015). Bright-field and dark-field images of a striatal section stained with DAB for MEnk are shown in Figures 2C,D, respectively. Contiguous sections stained for TH (Figures 2A,B) and MEnk (Figures 2C,D) show a correspondence between zones of low TH labeling and those of heightened MEnk labeling in both the CN and putamen.

**Figure 3:**
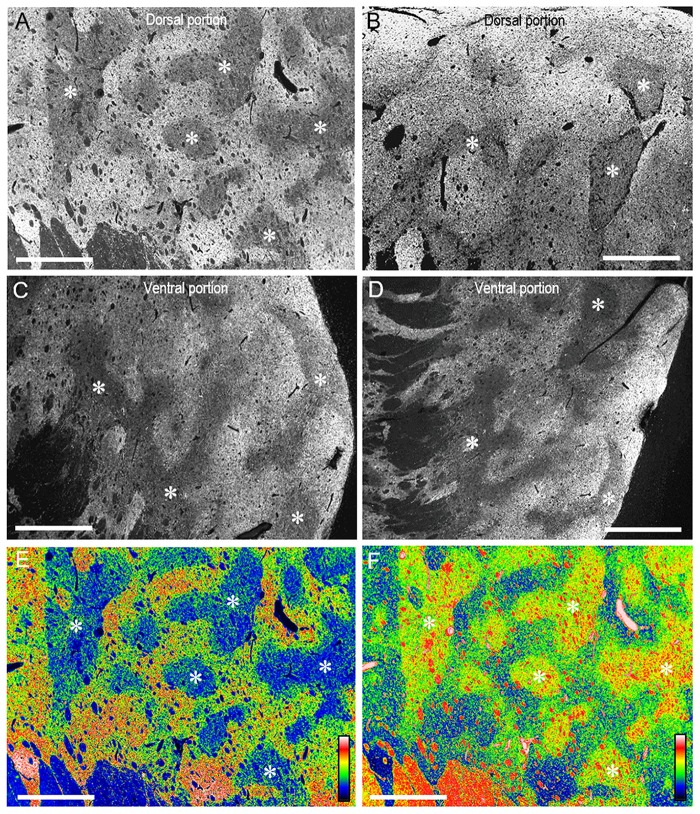
**Mosaic pattern of TH-immunostaining in the rostral putamen of the human brains. (A–D)** Photomicrographs of the dark-field images taken from the dorsal **(A,B)** and ventral portions **(C,D)** of the rostral putamen stained with DAB for TH. Asterisks indicate examples of striatal subfields with sparse TH labeling. **(E,F)** Graded color-converted representations of dark-field **(E)** and bright-field **(F)** images of the striatal area stained with DAB for TH, which is shown in **(A)**. A standard pseudocolor scale from blue (lowest level) through green, yellow, red, and white (highest level) was used. Asterisks indicate examples of corresponding zones poor in TH-labeling. Scale bars: 2 mm.

On dark-field microscopic images with low magnification, compartmental patterning of TH labeling was found in the rostral putamen (Figures 3A–D). A mosaic pattern of low- and high-immunoreactive zones that varied in their shapes and sizes was seen in both the dorsal (Figures 3A,B) and ventral (Figures 3C,D) portions of the rostral putamen. This was also evident in the graded color-converted images of dark-field (Figure 3E) and bright-field (Figure 3F) images of the putaminal area shown in Figure 3A.

**Figure 4:**
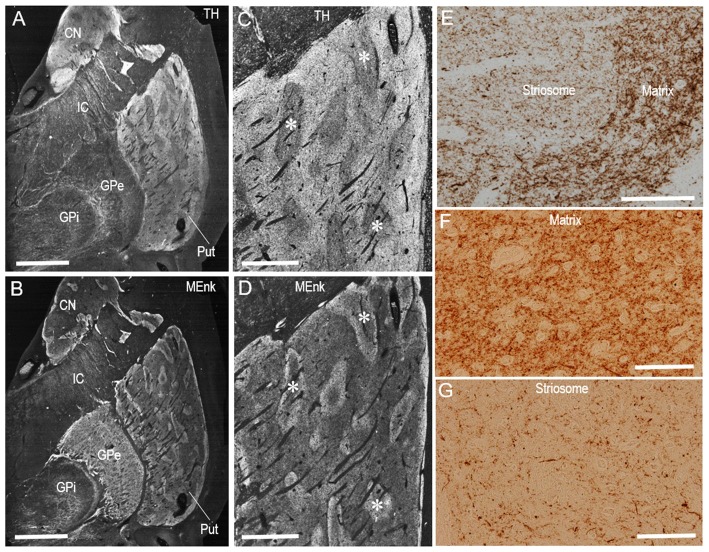
**Compartmental distribution of TH immunoreactivity in the caudal putamen of the human brains. (A–D)** Serial-section analysis of dark-field images of the lenticular nucleus **(A,B)** and caudal putamen **(C,D)** stained with DAB for TH **(A,C)** and MEnk **(B,D)**. Asterisks indicate examples of corresponding striosomes. **(E)** Photomicrograph at the border between the striosome and matrix in a putaminal area stained with DAB for TH. **(F,G)** Photomicrographs of the matrix **(F)** and striosome **(G)** in the putamen stained with DAB for TH. Note that the matrix area revealed a greater density of TH-positive fibers and puncta, compared with the striosomal area. Abbreviations: CN, caudate nucleus; Put, putamen; GPe, globus pallidus externa; GPi; globus pallidus interna; IC, internal capsule. Scale bars: **(A,B)** 5 mm, **(C,D)** 2 mm, **(E)** 500 μm, **(F,G)** 100 μm.

**Figure 5:**
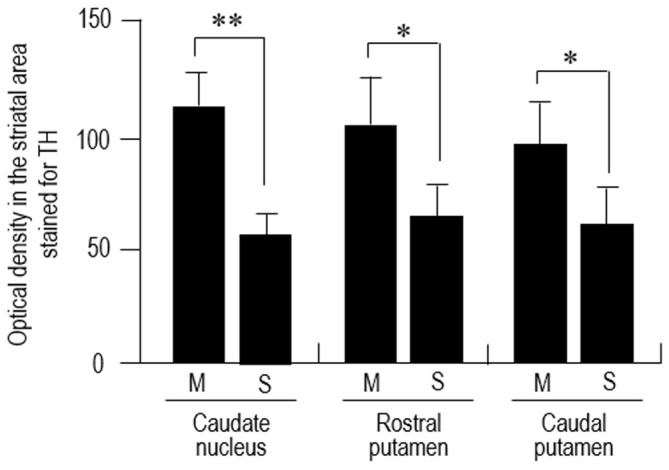
**Densitometric analysis of the human striatum stained for TH.** Measurements of the optical density of TH-immunoreactive DAB products were performed in the striosome (S) and matrix (M) compartments of the CN, rostral putamen and caudal putamen. Data represent means ± SEM (bars), *n* = 25. **indicates *P* = 0.01, M vs. S; *indicates *P* = 0.05, M vs. S.

Dark-field macroscopic images of contiguous sections of the lenticular nucleus stained with DAB for TH and MEnk are shown in Figures 4A,B, respectively. They represent the complementary distribution of TH and MEnk in the caudal portion of the putamen. Microscopic images with low magnification (Figures 4C,D) also show a striking correspondence between the TH-poor zones and the MEnk-rich striosomes. A photomicrograph of the border between the striosome and matrix areas (Figure 4E) shows less dense TH immunoreactivity in the striosome compared to the matrix. Higher-magnification images also demonstrate that the matrix area contains a greater density of TH-positive fibers and puncta (Figure 4F), compared with the striosomal area (Figure 4G). To confirm this visual impression, the optical density was measured on areas of the striosome and matrix in the CN, rostral putamen, and caudal putamen (Figure 5). The results showed that in all the striatal areas examined, the density of TH-labeling in the matrix was significantly higher than that in the striosomes.

### Putaminal Mosaic Visualized by TH-Immunofluorescence in Human Brains

**Figure 6:**
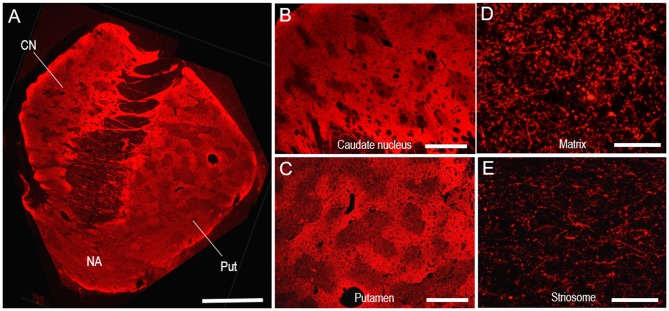
**Neostriatal mosaic visualized by TH-immunostaining with Cyanine 3 in human brains. (A)** A photomontage of a striatal section stained for TH. **(B,C)** Photomicrographs of the CN **(B)** and putamen **(C)** in the striatum stained for TH. **(D,E)** Photomicrographs of areas stained for TH in the matrix **(D)** and striosome **(E)** of the putamen. Abbreviations: CN, caudate nucleus; Put, putamen; NA, nucleus accumbens. Scale bars: **(A)** 5 mm, **(B,C)** 2 mm, **(D,E)** 50 μm.

Specific immunoreactivity for TH in the human neostriatum was also identified by our IHC protocols using fluorescence. In a photomontage of the striatum stained with Cyanine 3 for TH (Figure 6A), mosaic patterns were clearly seen in both the CN and putamen, as shown in Figure 2. Microscopic images with low magnification also revealed the compartmentalized distribution of TH labeling in the CN (Figure 6B) and putamen (Figure 6C). At higher-magnification, abundant TH-immunoreactive fibers and puncta were seen in the matrix (Figure 6D), but less so in the striosomes (Figure 6E).

**Figure 7:**
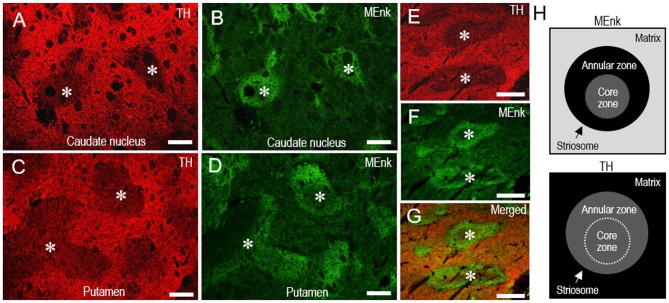
**Pattern of localization of TH in the striosome-matrix domains of the human neostriatum. (A–D)** Photomicrographs of the CN **(A,B)** and putamen **(C,D)** double-stained for TH **(A,C)** and MEnk **(B,D)**. Corresponding striosomes are indicated by asterisks. **(E–G)** Photomicrographs of an area of the putamen double-stained for TH **(E)** and MEnk **(F)**, with a merged image **(G)**. Corresponding striosomes are indicated by asterisks. Note that the margins of the TH-poor zones correspond to the outer margins of the striosomes enriched in MEnk. **(H)** Depicted are the patterns of localization of MEnk (*upper*) or TH (*lower*) in the striosome-matrix system. The striosome consists of annular and core zones. Higher densities of TH or MEnk-immunoreactive products are indicated by higher gray-scale levels. Scale bars: **(A–D)** 500 μm, **(E–G)** 250 μm.

Double immunofluorescence staining also revealed that TH immunoreactivity was sparse in the MEnk-enriched striosomes in the CN (Figures 7A,B) and putamen (Figures 7C,D). At higher-magnification, the margins of the TH-poor zones closely corresponded with the outer margins of the MEnk-rich zones (Figures 7E–G). Within the striosomes, there exist specialized zones rimming the striosomes, which are referred to as “annular” or “striocapsular” zones (Graybiel et al., 1981; Gerfen et al., 1985; Jakab et al., 1996; Holt et al., 1997; Brimblecombe and Cragg, 2015). These express higher levels of MEnk than the core (center) zones, as depicted in Figure 7H. Based on the findings shown here, it is likely that TH immunoreactivity is diffusely distributed within the striosomes, with no apparent difference in the TH-staining intensity between the annular and core zones (Figure 7H).

**Figure 8:**
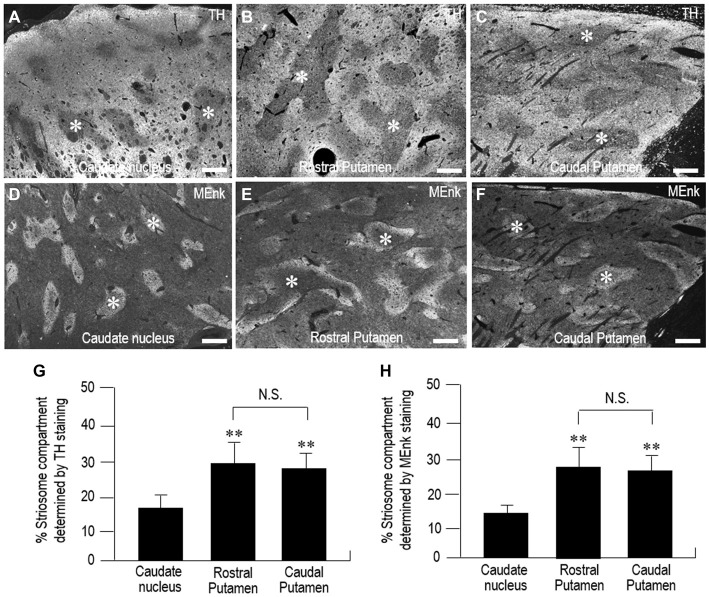
**Morphometric analysis of the percentage of the striatum occupied by the striosome compartment in the human neostriatum. (A–F)** Gray-scale-converted microscopic images of striatal areas stained with fluorescence for TH **(A–C)** and MEnk **(D–F)** in the CN **(A,D)**, rostral putamen **(B,E)**, and caudal putamen **(C,F)**. Asterisks indicate examples of striosomes. Scale bars: 1 mm. **(G)** Measurements of the percentage of the striatum occupied by the striosome compartment identified by TH-immunostaining in the CN, rostral putamen, and caudal putamen. Data represent means ± SEM (bars), *n* = 25. **indicates *P* < 0.01 vs. CN; N.S. (not significant) indicates *P* > 0.05, Rostral Putamen vs. Caudal Putamen. **(H)** Measurements of the percentage of the striatum occupied by the striosome compartment identified by MEnk-immunostaining in the CN, rostral putamen, and caudal putamen. Data represent means ± SEM (bars), *n* = 25. **indicates *P* < 0.01 vs. CN; N.S. (not significant) indicates *P* > 0.05, Rostral Putamen vs. Caudal Putamen.

Representative gray-scale images of the striatal areas stained for TH (Figures 8A–C) and MEnk (Figures 8D–F) are shown for the CN (Figures 8A,D), rostral putamen (Figures 8B,E), and caudal putamen (Figures 8C,F). These images (also see, Figures 2, 6) reveal an apparent difference in the striosome-to-matrix ratio among striatal areas. To confirm this visual impression, we carried out a morphometric analysis of the striatal sections. In the striatum stained for TH (Figure 8G), the percentage of the striatum occupied by the striosome compartment in the CN (18.2% ± 3.2%) was significantly different (*P* < 0.05) from that in both the rostral putamen (29.8% ± 6.7%) and caudal putamen (28.1% ± 5.2%). In the striatum stained for MEnk (Figure 8H), the percentage of the striatum occupied by the striosome compartment in the CN (14.0% ± 2.1%) was significantly different (*P* < 0.05) from that in both the rostral putamen (29.2% ± 6.4%) and caudal putamen (28.1% ± 5.1%). Thus, the striosome-to-matrix ratio of the putamen was 1.5~2.0 times that of the CN.

### Sparse Distribution of TH-Expressing Interneurons in the Human Neostriatum

**Figure 9:**
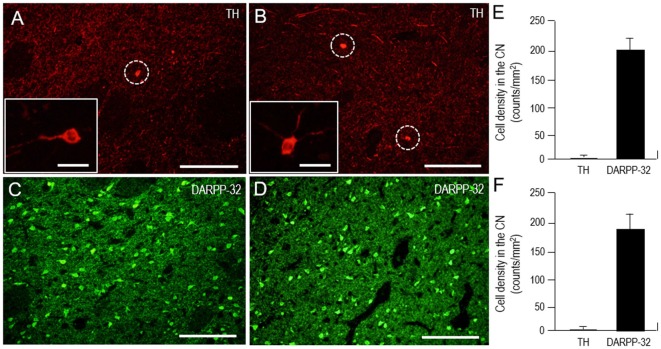
**Scarcity of TH-positive interneurons in the human neostriatum. (A–D)** Photomicrographs of the CN **(A,C)** and putamen **(B,D)** stained for TH **(A,B)** and DARPP-32 **(C,D)**. The TH-positive cells are indicated by dashed open circles **(A,B)**. Higher-magnification images of the TH-positive cells are also shown in the *insets* in **(A,B)**. Scale bars: **(A–D)** 200 μm, (*insets* in **A** and **B**) 20 μm. **(E,F)** Cell density measurements of striatal cells positive for TH or dopamine and cAMP-regulated phosphoprotein of 32 kDa (DARPP-32) in the CN **(E)** and putamen **(F)**. Values are means ± SEM (bars), *n* = 25.

It is known that the mammalian striatum contains a distinguished subgroup of GABAergic interneurons that express TH (for review see, Silberberg and Bolam, 2015). In this study, we also identified the TH-positive cells in both the CN and putamen (Figure 9). However, cell density analysis (Figures 9E,F) revealed that the TH cells were only sparsely distributed in the CN (1.8 ± 1.1 cells/mm^2^) and putamen (1.6 ± 1.0 cells/mm^2^), while striatal cells positive for DARPP-32, a marker for medium spiny neurons (Okita et al., 2012), were abundantly found in the CN (201 ± 22 cells/mm^2^) and putamen (192 ± 31 cells/mm^2^).

## Discussion

In this study, we showed an immunohistochemical evidence that TH is compartmentally distributed in both the CN and putamen in FFPE human autopsy brains. By means of the serial section analysis and the double fluorescent labeling technique, the present study indicates that TH is enriched in the matrix compartment relative to the striosomes at the striatal levels from the rostral to the caudal throughout. We suggest that in the entire neostriatum, TH immunoreactivity forms the distinct striosome-matrix configurations in the same manner. This notion may contradict the previous report indicating that in the human striatum, striosomes are largely devoid of TH at rostral striatal levels while the inverse is found caudally (Prensa et al., 1999).

TH is highly concentrated in the neostriatum, where nearly all of it is expressed in axons of the dopaminergic nigrostriatal afferents. However, it has so far been known that in the mammalian striatum, there exists a subset of GABAergic interneurons that express TH (Silberberg and Bolam, 2015), although their roles in striatal dopamine systems have not yet been determined (Xenias et al., 2015). This means that striatal TH labeling is composed of not only the TH-positive afferents arising from the SNc but also the TH-immunoreactive products of such interneurons. Indeed, we here identified the TH-positive cells in the human neostriatum (Figure 9). In agreement with the previous report (Bernácer et al., 2012), our results, however, documented that the TH cells were only scarcely distributed in both the CN and putamen. The sparse density of the TH-positive cells casts doubts about their contribution to the mosaic organization of TH labeling in the human neostriatum.

Among striatal neurons, striosomal cells are unique because they send their GABAergic inhibitory projections directly or indirectly to the SNc, which contains dopaminergic neurons that project back to both the striosome and matrix compartments (Gerfen, 1984; Jiménez-Castellanos and Graybiel, 1989; Tokuno et al., 2002; Fujiyama et al., 2011; Watabe-Uchida et al., 2012). Accordingly, the striosome compartment could exert global control over striatal dopamine signals by inhibiting the activity of nigral dopaminergic cells (Crittenden and Graybiel, 2011). Considering possible mechanisms that underlie the inhomogeneous organization of TH immunoreactivity in the striatum of mature human brains, Graybiel et al. (1987) suggested an involvement of presynaptic differences between the striosome and matrix compartments in enzymatic regulation of dopamine content. We here showed that in the neostriatal sections stained for TH, the striosome-to-matrix ratio of the putamen is significantly higher (*P* < 0.05) than that of the CN (Figure 8). Taken together, we suggest that there might be a difference between the CN and putamen in the compartment-specific homeostatic regulation of striatal dopaminergic activities.

Given the evidence the patch (striosome) and matrix compartments comprised about 15% and 85% of the striatal volume, respectively, in the different species that included rat, rhesus monkey, and human, Johnston et al. (1990) suggested that a relatively constant ratio of 15% patch to 85% matrix area is maintained across mammalian species including humans. However, they also noted a slight, but significant increase (1.7 times) in the percentage of the striatum occupied by the striosome compartment from rat, through rhesus monkey, to human striatum (Johnston et al., 1990). Moreover, they also described that averaged across species, the rostral striatal sections were slightly richer in patch compartment (17%) than the caudal sections (9.8%) (Johnston et al., 1990). Our present study showed that in the human neostriatal sections stained for MEnk, the percentage of the striatum occupied by the striosome compartment in the CN (14.0%) was significantly different (*P* < 0.05) from that in both the rostral putamen (29.2%) and caudal putamen (28.1%). This indicates that the striosome-to-matrix ratio of the putamen was about 2.0 times that of the CN in the human neostriatum. In view of these observations, we posit that a species and regional difference of the small degree in the striosome-to-matrix ratio could be found in the striatum of mammals.

The putamen plays a critical role in the basal ganglia-thalamocortical circuit that regulates our voluntary movements and motor learning. Further anatomical and physiological research on the putamen is, therefore, important for elucidating the pathogenesis and symptomatology of human movement disorders. The putamen also serves as the most suitable site for the intrastriatal application of dopamine-producing transplants in PD patients (Lindvall, 2015), as previously suggested (Kish et al., 1988). There is a growing body of evidence that striosome-matrix dopaminergic systems might be involved in the disease-specific striatal pathology and symptoms of multiple human movement disorders (Crittenden and Graybiel, 2011). Indeed, human postmortem analyses have shown compartment-specific pathology in the striatum of patients with movement disorders, such as Huntington’s disease (OMIM143100; Ferrante et al., 1987; Goto et al., 1989b; Goto and Hirano, 1990a; Morton et al., 1993; Hedreen and Folstein, 1995; Tippett et al., 2007), multiple system atrophy (OMIM146500) of the parkinsonian type (Goto and Hirano, 1990b; Sato et al., 2007), and X-linked dystonia-parkinsonism (OMIM314250; Goto et al., 2005, 2013). In this study, we performed an IHC study on FFPE tissues from autopsied human brains, and disclosed that striatal dopaminergic innervations labeled for TH are organized into discrete striosome-matrix configurations in the putamen. Our present findings provide anatomical evidence supporting the intriguing concept that compartment-specific dysfunction of dopamine-related neural circuits might underlie the genesis of human movement disorders.

## Author Contributions

SG contributes to the conception or design of the work; and the acquisition, analysis, and interpretation of data for the work. SG wrote the manuscript. RM contributes to the conception or design of the work; and the acquisition, analysis, and interpretation of data for the work.

## Conflict of Interest Statement

The authors declare that the research was conducted in the absence of any commercial or financial relationships that could be construed as a potential conflict of interest.
